# The Association between Systemic Glucocorticoid Use and the Risk of Cataract and Glaucoma in Patients with Rheumatoid Arthritis: A Systematic Review and Meta-Analysis

**DOI:** 10.1371/journal.pone.0166468

**Published:** 2016-11-15

**Authors:** Rachel J. Black, Catherine L. Hill, Susan Lester, William G. Dixon

**Affiliations:** 1 Arthritis Research UK Centre for Epidemiology, Manchester Academic Health Sciences Centre, The University of Manchester, Manchester, United Kingdom; 2 Discipline of Medicine, The University of Adelaide, Adelaide, Australia; 3 Rheumatology Unit, The Queen Elizabeth Hospital, Woodville, Australia; 4 Health e-Research Centre, Farr Institute for Health Informatics Research, Manchester Academic Health Sciences Centre, The University of Manchester, Manchester, United Kingdom; 5 Department of Rheumatology, Salford Royal NHS Foundation Trust, Salford, United Kingdom; University of Birmingham, UNITED KINGDOM

## Abstract

**Objective:**

Glucocorticoids (GCs) are often used to treat Rheumatoid Arthritis (RA) despite their many side effects and the availability of other effective therapies. Cataract and glaucoma are known side effects of GCs but the risk of them developing in the setting of GC use for RA is unknown. The aim was to perform a systematic review and meta-analysis to determine the association between GCs and the risk of developing cataract and/or glaucoma in RA.

**Methods:**

A systematic search was carried out using MEDLINE, EMBASE, and Web of Science. All RCTs comparing GC use to non-use in RA populations were sought. Observational studies reporting cataract and/or glaucoma amongst GC users and non-users were also included. Data extracted included incidence/prevalence of cataract and/or glaucoma in each arm, dose and duration of therapy. Two independent reviewers performed quality assessment.

**Results:**

28 RCTs met eligibility criteria, however only 3 reported cataracts and glaucoma, suggesting significant under-reporting. An association between GC use and the development of cataracts in RA patients was seen in observational studies but not RCTs. There was no statistically significant association between GC use and the development of glaucoma, although data were sparse. There were insufficient data to determine the impact of dose and duration of therapy.

**Conclusion:**

The current literature suggests a possible association between GC use and the development of cataract. However, this risk cannot be accurately quantified in RA from the available evidence. RCTs have not adequately captured these outcomes and well-designed observational research is required.

## Introduction

Cataract and glaucoma were first described as side effects of systemic GC therapy as early as 1953[1, 2]. In particular, posterior subcapsular cataracts (PSCs) are a subtype of cataracts that occur more frequently in GC-exposed patients [1]. Similarly, GC exposure can lead to steroid-induced glaucoma, a type of open angle glaucoma. These conditions can lead to visual impairment, resulting in significant disability and cost to the healthcare system [3]. As GCs remain widely prescribed in rheumatoid arthritis (RA) and many other inflammatory diseases [4, 5], any increased risk might lead to a significant public health burden.

EULAR guidelines advocate that clinicians inform their patients of the risk of side effects associated with GCs before commencing treatment [6]. However, for cataract and glaucoma, the magnitude of risk is rarely reported and current literature has not been reviewed to determine if the risk can be accurately quantified. Specific questions, such as how this is influenced by dose and duration of therapy, also have not yet been addressed. RA was the first condition to be treated with GCs in 1948 [7] and it was also the first condition in which PSCs were described as AEs of GC use in 1950 [1]. There are multiple randomised controlled trials (RCTs) of GC use in RA, and long-term use has been looked at in many observational studies, making it an ideal setting to explore these questions.

The aim was to perform a systematic literature review and meta-analysis of RCTs and observational studies examining the association between GC use and the risk of cataract and glaucoma in patients with RA compared to patients with RA not exposed to GCs. Secondary aims were to determine whether there is an association with dose and duration of GC therapy.

## Materials and Methods

### Search strategy

A systematic search was conducted using MEDLINE, EMBASE and Web of Science for articles published to January 2016. There was no review protocol for this systematic review. Separate search strategies were conducted for RCTs and observational studies. RCTs fulfilling the following criteria were included: 1) RA population 2) Exposure to systemic (oral, intramuscular or intravenous) GC therapy in one arm and non-exposure (no treatment or placebo) in at least one comparator arm. Studies comparing DMARD(s) plus GC in one arm and the same DMARD(s) without GC in the comparison arm were also included. Inclusion criteria for observational studies were: 1) RA population, 2) Use of a cross-sectional, case-control or cohort study design 3) Reporting of the number or rate of cataracts and/or glaucoma in GC-exposed and non-exposed patients. Although cross-sectional studies do not provide information on causality, they were included in this review in order to capture information from about PSCs from early studies conducted in the 1960s. Studies conducted exclusively in populations other than RA (including early inflammatory arthritis, undifferentiated polyarthritis and JIA) were excluded. If RA was one of several indications reported, the study was only included if the RA population was reported separately from the other populations. Exposure was restricted to systemic GC therapy (oral, intramuscular, intravenous), and studies reporting only topical, intra-articular, intra-ocular or other non-systemic routes of steroid administration were excluded. In instances where two studies reported on the same cohort, the earlier of the two studies was retained unless the latter reported on cataracts and/or glaucoma.

Search terms are listed in the online supporting information (S1 Table). Search filters for RCTs were based on the Cochrane Highly Sensitive Search Strategy for identifying randomized trials in MEDLINE: sensitivity-maximizing version [8].The search filters for observational studies was based on the Scottish Intercollegiate Guidelines Network search filters for observational studies [9].

### Study Selection and Data extraction

The initial study selection was based on review of title and abstract. Studies in non-RA populations were excluded at this stage, as were those with designs other than RCTs, cohort, case control or cross-sectional studies. Only articles published in English were selected due to lack of access to a translation service. Full text manuscripts of all remaining articles were reviewed for eligibility based on the inclusion and exclusion criteria. Hand searching of references of all papers obtained through the above search and relevant review articles was also carried out. Abstract only publications and unpublished studies were not considered. A random sample of 20 RCTs was selected for independent full-text review by the second reviewer (CH). Each study was assessed in regards to eligibility criteria and the rate of agreement with the first reviewer (RB) was then determined.

Data extraction was performed by a single reviewer using an electronic data collection form. Information was extracted regarding: 1. The RA population including age, gender and disease duration, 2. The exposure including the definition of GC use, dose and duration of therapy, 3. The comparator arm including whether this was placebo or non-GC exposure, and 4. The outcome of cataract and glaucoma in each arm, how this was obtained and whether this was reported as point prevalence, prevalence, incidence or incidence rate.

### Quality Appraisal

Both reviewers (RB, CH) performed an independent assessment of the quality of all included studies. Discrepancies were discussed and resolved by consensus. The Cochrane Risk of Bias Assessment tool [10] was used to assess the quality of all included RCTs reporting cataract and/or glaucoma. Cohort studies were assessed using the Newcastle Ottawa Scale[11] and each of the cross-sectional studies was assessed for selection bias, detection bias, attrition bias, and reporting bias, based on the Cochrane Handbook common classification scheme for bias[12]. No case control studies meeting eligibility criteria were identified in the search. The RCTs and cross-sectional studies were assessed as low, high or uncertain risk, however the cohort studies were assessed as either being low or high risk due to differences in the scales used.

### Meta analysis

Although data were limited, meta-analyses were performed as a means of summarising the data available. Cross sectional studies were not considered in the meta-analysis due to their inability to assess causality. Random effects meta-analyses were performed for each outcome: all cataracts and glaucoma. While other methodologies for dealing with zero events and sparse data were considered [13–15], risk difference (RD) was selected as the effect size because many studies had zero events in one arm, and odds ratios and relative risks are not defined in this setting. Statistical heterogeneity was assessed using the I^2^ statistic, where I^2^>50% represents significant heterogeneity. Meta analyses were carried out using the Metafor package in R [16].

### Effect of dose and duration of GC exposure and visual outcomes

A description, rather than formals analysis of the effect of dose and duration of GC use on the development of cataract and glaucoma was carried out due to the scarcity of studies addressing this.

### Comparing the reporting of cataracts in RCTs, to population-based incidence of cataracts

Concerns that cataracts were not being captured or reported in RCTs, early in the course of this review, led to a secondary aim to compare the RCT incidence with the expected incidence of cataracts based on general population rates derived from The Blue Mountains Eye Study (BMES) [17]. For comparison with RA RCTs, the expected population cumulative incidence of cataracts over 1 and 2 years was estimated for patients aged less than 75 years from the reported BMES 5-year incidence, under the constant hazards assumption.

## Results

### Randomised Controlled Trials

**Fig 1:**
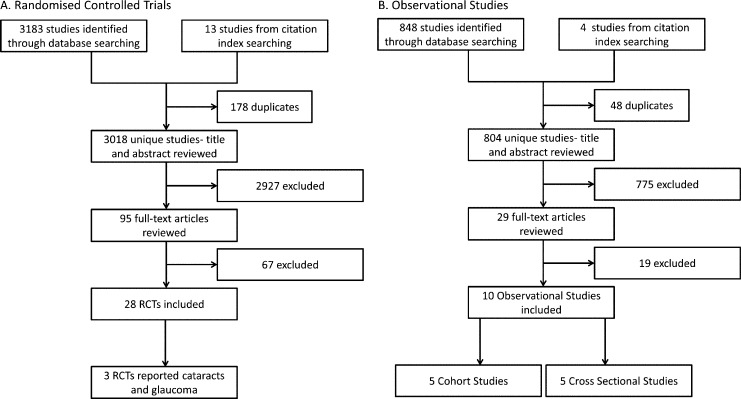
Flow charts depicting selection of A. Randomised Controlled Trials and B. Observational Studies.

95 RCTs were identified for full-text review and 28 RCTs were ultimately included after applying eligibility criteria (Fig 1). There was initial agreement for 19/20 articles selected for assessment by the second reviewer. Disagreement occurred for one article because the results of this study had already been reported earlier in another paper that had not been included in the random sample. Once this issue was made known to the second reviewer, there was full consensus for all articles. These papers are summarised in the supporting information (S2 Table).

**Fig 2:**
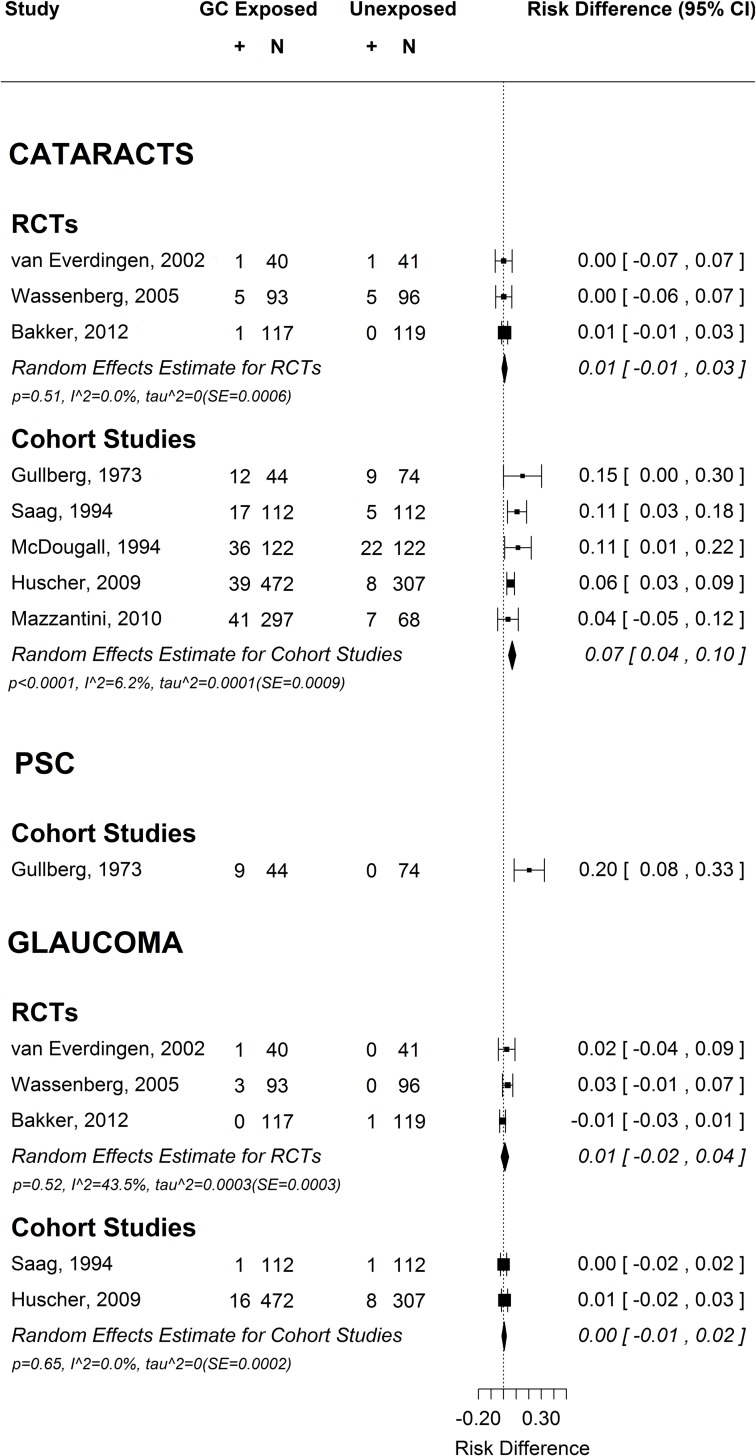
Meta analysis forest plots of RCTs and cohort studies reporting all cataracts, cohort studies reporting PSCs and RCTs and cohort studies reporting glaucoma.

Cataracts and glaucoma were reported in only 3 of the 28 RCTs (Table 1), each of 2 years’ duration. The initial 28 RCTs evaluated treatment with oral, intramuscular and intravenous GCs, however the final three studies all assessed oral exposure. These three studies reported all cataracts rather than the PSC subtype. One study, Williams et al [18], was excluded because it did not report whether the single observed glaucoma case occurred in the GC or control group. The remaining 24 RCTs did not mention cataracts and/or glaucoma in either the methods or the results. The protocols for all three RCTs reporting incident eye disease included either recording specific known GC-associated AEs using a standardised list [19, 20] or ophthalmological examinations [20, 21]. There were no significant differences in the risk of developing cataracts for GC-exposed versus unexposed RA patients, either individually by trial, or collectively in the meta-analysis (RD 0.01 events/patient, 95% CI -0.01–0.03), Fig 2 (A RD of 0.01 equates to an additional 10 cataracts for every 1000 patients exposed to GCs compared to those unexposed). Similarly, there were no significant differences in the risk of developing glaucoma for GC-exposed versus unexposed RA patients, either individually by trial, or collectively in the meta-analysis (RD 0.01 events/patient, 95% CI -0.02–0.04), Fig 2.

**Table 1:** RCTs comparing GC use to non-use in RA and number of reported cataracts and glaucoma.

First Author,	Duration of study	Country	Mean Age (%Female)	Arms of RCTs (n)	Outcome Reported & method of detection
Year					
Bakker [19],	2 years	The Netherlands	54	MTX + PNL 10mg (117)	Incident cataract and glaucoma-standard list of AEs
2012			(60%)	MTX + placebo (119)	
van Everdingen [20],	2 years	The Netherlands	62	PNL 10mg (40)	Incident cataract and glaucoma- standardised list of AEs, ophthalmologic expertise requested when necessary
2002			(64%)	Placebo (41)	
Wassenberg [21],	2 years	Germany, Austria, Switzerland	52	Gold or MTX + PNL 5mg (93)	Incident cataract and glaucoma- ophthalmologic exams at the beginning and end of the study
2005			(70%)	Gold or MTX + placebo (96)	

RCTs = Randomised Controlled Trials, GC = glucocorticoid, RA = rheumatoid arthritis, n = number, PNL = prednisolone, MTX = methotrexate, AEs = Adverse Events

### Comparing the reporting of cataracts in RCTs to population-based incidence of cataracts

Of the 28 RCTS comparing GC use to non-use in RA, there were a total of 13 cataracts reported in only 3/28 studies (11%). The remaining 25 studies did not report cataracts in their results, nor did they set out to do so in the their methods. Across all 28 RCTs there was a total of 4604 person years, giving a combined cataract incidence rate of 2.8 per 1000 person years (pyr).

Considering incidence only in the three studies that reported cataracts, the random effects estimate of the combined incidence rate (IR) was 11/1000pyr (95%CI 3-43/1000pyr), although the IRs ranged considerably from 2.1/1000pyr [19] to 12.3/1000pyr [20] to 26.5/1000pyr [21]. This heterogeneity is likely related to the different methods used to detect cataract, with ophthalmological examination detecting a greater prevalence of cataracts.

The cumulative two-year incidence in the 3 studies of this duration reporting cataract was 2.0% (95% CI 0.6–6.6). The expected population cumulative incidence (in participants younger than 75 years), estimated from the BMES population [17], is 6% over 1 year and 12% over two years. Given that the median duration of the 27 RCTs was one year, this suggests that cataracts were expected in RCTS in which they were not reported, suggesting a substantial six-fold under-reporting of cataracts in the clinical trials.

### Observational studies

A total of 10 observational studies met eligibility criteria for the final review. Five were cohort studies, of which two were prospective and three retrospective. The remaining five were cross-sectional (Fig 1).

#### a) All Cataracts (Reported in 5 cohort studies)

Amongst the cohort studies, Gullberg *et al* [22] reported PSCs as well as all cataracts. However, the remaining cohort studies reported all cataracts only and not PSCs [23–26]. The methods for identifying and defining cataracts varied between studies, as did the definitions of GC use (Table 2).

**Table 2:** Observational Studies comparing rates of cataracts amongst GC uses and non-users.

First Author, Year	Study Design, Outcome reported (number patients)	Age	Gender (Percentage Female)	RA disease duration	Definition of GC use	Duration on GCs	Prednisolone equivalent dose/d	Cataract Type and Method of Detection
**Black [1],**	Cross-sectional,	<30 = 3, 30–39 = 9,	37/67	1-3y = 20,	Not defined	<1y = 9,	<10mg = 6,	PSC
**1960**		40–49 = 24,	(55%)	4-6y = 13,		1-4y = 22,	10-15mg = 22,	Ophthalmologist
	Point prevalence	50–59 = 16,		7-9y = 12,		>4y = 13	>15mg = 16	
		≥60 = 11		>9y = 18				
	(63)							
**Giles [27],**	Cross-sectional,	<30 = 3,	45/62	1-3y = 15,	Sustained GC	<1y = 3,	<10mg = 19,	PSC
**1962**		30–39 = 8,	(73%)	4-6y = 12,	use for	1-4y = 18,	10-15mg = 12,	Ophthalmologist
	Point prevalence	40–49 = 20,		7-9y = 11,	≥6months	>4y = 17	>15mg = 7	
		50–59 = 26,		>9y = 24				
	(62)	≥60 = 5						
**Crews [28],**	Cross-sectional,	**Patients with PSC**	Not Reported	Range 3-38y	Being treated with GCs	**Patients with**	**Patients with**	PSC
**1963**		40–49 = 2, 50–59 = 7,				**PSC**	**PSC**	Ophthalmologist
	Point prevalence	60–69 = 8, 70–79 = 1				Mean 4.5y	Mean 10.5mg	
		**Without PSC**				**Without PSC**	**Without PSC**	
	(86)	Not Reported				Not reported	Not reported	
**Furst [29],**	Cross-sectional,	Not Reported	Not Reported	Not reported	GC use for	**Patients with**	**Patients with**	PSC
**1966**					minimum of	**PSC**	**PSC**	Ophthalmologist
	Point prevalence				1year	Mean 4.5y	Mean 12.2mg	
						**Without PSC**	**Without PSC**	
	(105)					Mean 4.75y	Mean 9.5mg	
**Williamson [30],**	Cross-sectional*,	**GC-users**	243/307	**GC-users**	Not defined	Mean 3.15y	Mean	PSC
**1969**		Mean 52.5	(79%)	Mean 8.95y		SD +/-2.45	10.89mg	Ophthalmologist
	Point prevalence	SD +/-11.5		SD +/- 6.35			SD +/-4.8	
		Range 18–80		**Non-users**				
	(307)	**Non-users**		Mean 6.55y				
		Mean 49.3		SD +/-5.9				
		SD +/-15						
		Range 18–83						
**Gullberg [22],**	Retrospective cohort,	Mean 58	100/130	Mean 11 y	Not defined	**Patients with**	**Patients with**	PSC and all
**1973**		Range 18–80	(77%)	Range <1-38y		**PSC**	**PSC**	cataracts
	Prevalence					Mean 1640d	Mean 7.6mg	Ophthalmologist
	(118)					**Without PSC**	**Without PSC**	
						Mean 1360d	Mean 7.0mg	
**McDougall [24],**	Prospective cohort,	**GC-users**	170/244	**GC-users**	Received GC	Mean 6.9y	Mean 8.0mg	Not Stated
**1994**		Mean 55.9	(70%)	Mean 14.1y	after study	Range 0.3–21.8y	Range 1.0-	Rheumatologist
	Prevalence	Range 17.1–81.3		**Non-users**	enrolment		23.4	ophthalmoscope
	(244)	**Non-users**		Mean 13.8y	(excluded if			examination at each
		Mean 56.0			prior			visit, ophthalmology
		Range 18.1–82.0			exposure)			referral if needed
**Saag [23],**	Retrospective cohort,	**GC-users**	168/224	**GC-users**	Near	Not reported	Mean 6.1mg	Not Stated
**1994**		Mean 51.8	(75%)	Mean 4.9y	continuous		SD +/-3.1mg	Clinical records
	Incidence	SD +/-12.7		SD+/-6.3,	(<1mth off)			
	(224)	**Non-users**		**Non-users**	steroids			
		Mean 51.7		Mean4.9y	for>1y at			
		SD +/-12.5		SD +/-6.7	≤15mg/d			
**Huscher [25],**	Prospective cohort,	No GC = 58.4	No GC = 79%	No GC = 10.8y,	Any GC use in	Not reported	<5mg = 101,	Not Stated
**2009**		<5mg = 60.9	<5mg = 82%	<5mg = 9.7y,	the past 12m		5–7.5mg = 281,	Self reported
	Point prevalence	5–7.5mg = 60.4	5–7.5mg = 77%	5–7.5mg = 13.2y,	for >6m		>7.5mg = 90	
		>7.5mg = 61.5	>7.5mg = 72%	>7.5mg = 10.7y				
	(779)							
**Mazzantini [26],**	Retrospective cohort,	**GC-users**	**GC-users**	**GC-users**	GC use	Mean 8y	Mean 4.1mg	Not Stated
**2010**		Mean 66.7	65%	Mean 16.8y	continuously	SD +/-6y	SD+/-1.2mg	Clinical records
	Prevalence	SD +/-11.7	**Non-users**	SD +/-6.3y	for >6m and	Range 1-20y	Range 4-6mg	
	(365)	Range 26–89	74%	**Non-users**	<10y			
		**Non-users**		Mean 16.3y				
		Mean 66.1		SD +/-6.1y				
		SD+/-12.3						
		Range 28–90						

*Some patients followed up prospectively with repeat ophthalmological examinations performed two or more times at 3 or 6 months intervals.

n = number, PSC = posterior supcapsular cataract, GC = glucocorticoid, d = day, m = month, y = year, SD = standard deviation

In contrast to RCTs, four of the five cohort studies reported a significantly increased risk of cataracts in GC-exposed patients, and collectively the combined risk difference was 0.07 events per person (95%CI 0.04–0.10), Fig 2, which is equivalent to an additional 70 cataracts seen per 1000 patients in those exposed to GCs. The odds ratio, which could also be meaningfully estimated from this data, was 2.1 (95%CI 1.5–2.9). While there was considerable variation in age, disease duration, dose and duration on GCs between these studies (Table 2), there was surprisingly little statistical evidence of heterogeneity between the effect sizes (RD I^2^ = 6.2%).

In regards to dose and duration of therapy, Huscher *et al* [25] looked at patterns of glucocorticoid induced side effects and found two distinct patterns including ‘linear risk’, where risk of developing an AE increased with dose in a linear fashion and ‘threshold risk’ where the increased risk only occurred after a threshold dose was reached. They found that both cataract and glaucoma fit the threshold pattern with a threshold dose of 5mg per day for cataract and 7.5mg per day for glaucoma in patients on GCs for more than 6 months. Mazzantini *et al* [26] did not find any difference in cataract prevalence between those treated with GCs for <2 years, 2–5 years or >5years.

#### b) Posterior Supcapsular Cataracts (PSCs) (Reported in 5 cross-sectional studies and 1 cohort study)

PSCs were reported in five cross-sectional studies published between 1960 and 1969. In all five studies, an RA population, including those exposed and unexposed to GC treatment, underwent ophthalmological examination. In 4/5 of these studies[1, 27–29], PSCs were only seen in those exposed to GCs, and in the remaining study by Williamson et al [30], 1 PSC was seen amongst 159 unexposed to GCs compared to 10/148 exposed (Table 3). PSCs were also reported in a retrospective cohort study by Gullberg et al [22], with a risk difference of 0.20 events per patient (95%CI 0.08–0.33), equivalent to an additional 200 PSCs for every 1000 patients exposed to GCs.

**Table 3:** Cross-sectional Studies Reporting Posterior Subcapsular Cataracts (PSCs).

Study	GC-Exposed		GC-Unexposed	
Author, Year	Number of patients	Number of PSCs	Number of patients	Number of PSCs
**Black [1], 1960**	44	17	19	0
**Giles [27], 1962**	38	14	24	0
**Crews [28], 1963**	52	18	34	0
**Furst [29], 1966**	57	6	48	0
**Williamson [30], 1969**	148	10	159	1

GC = glucocorticoid, PSCs = Posterior Subcapsular Cataracts

In regards to dose and duration of GC therapy, Black *et al* reported that PSCs were not seen in six patients who took prednisolone equivalent doses of <10mg per day or in nine patients who had been taking GCs for less than one year [1]. Giles *et al* [27] also found that PSCs did not occur in three patients that received GCS for less than one year. However they did occur in four of the 19 patients taking <10mg per day for ≥6 months. The prevalence of PSCs increased with GC dose, with PSCs seen in 4/12 patients on 10-15mg/day and in 6/7 patients on >15mg per day. Furst *et al* compared the prednisolone equivalent average daily dose and duration of therapy in those with PSCs to those without and found no significant difference in duration of therapy but a higher average dose of 11.5mg in those that developed PSCs compared to 8.8mg in those that did not. However, they did not report whether this difference was significant. Williamson also reported an increased prevalence with longer duration of therapy, with no PSCs seen in the 65 patients on GCs for less that two years compared to seven of the 52 patients on GCs for more than two years (p<0.01) [30].

#### c) Glaucoma (Reported in2 cohort studies and 1 cross-sectional study)

Of the observational studies that fulfilled the eligibility criteria for this search, three studies reported glaucoma [23, 25, 30]. No significant association was seen between GC use and glaucoma amongst the 2 cohort studies [23, 25] with a combined risk difference of 0.00 (95% CI -0.01–0.02). In the cross-sectional study, Williamson *et al* [30] reported 1 case of glaucoma amongst 148 GC users and 1 case amongst 159 non-users.

### Quality Assessment

**Fig 3:**
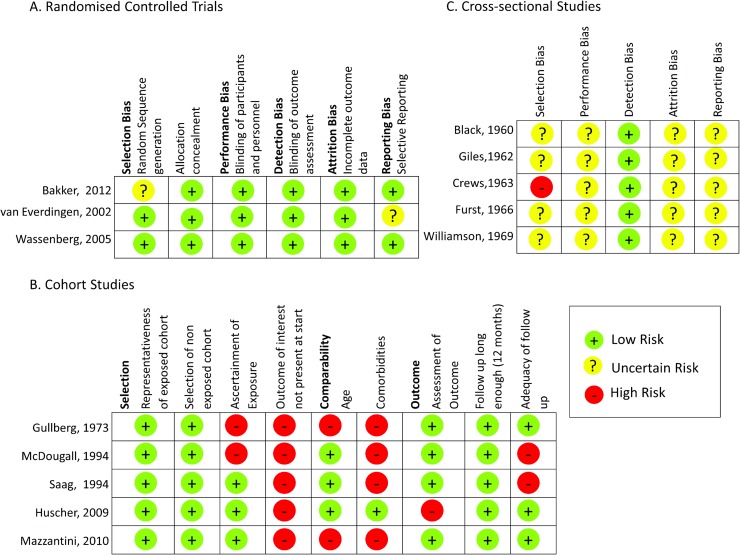
Quality Assessment of A. Randomised controlled trials and C. Cross sectional studies using the Cochrane Risk of Bias Assessment Tool (high risk, low risk of uncertain risk) and B. Cohort studies using the Newcastle Ottawa Scale (high risk or low risk).

The quality assessment of all studies is shown in Fig 3. Among RCTs, the risk of bias was low for most criteria (Fig 3A). Conversely, for the cohort studies, there was a high risk of bias in some categories, particularly regarding the exclusion of prevalent eye disease (Fig 3B). Amongst the cross-sectional studies, the risk of bias was uncertain for most categories (Fig 3C).

## Discussion

RA is an ideal patient group in which to look for long term adverse effects of GCs. RA was the first condition to be treated with GCs [7]and it was also the first condition in which PSCs were described as AEs of GC use [1]. More recently GCs have been shown to have a disease modifying effect in RA [31], resulting in more continuous patterns of use compared to other indications such as asthma and IBD for which GCs are often used intermittently. RA patients may differ in their risk of developing GC induced eye diseases compared to other populations such as those with IBD and spondyloarthropathies, which are more frequently associated with uveitis and topical GC use, other known risk factors for developing cataract and glaucoma [32]. It is therefore important to understand how systemic GC use affects the risk of developing cataract and glaucoma in RA patients.

This review has found that there is limited literature addressing the association between systemic glucocorticoid use and the risk of cataract and glaucoma in patients with RA. In contrast to the RCTs which showed no association between GC use and all cataracts (RD 0.01, 95%CI -0.01–0.03), an association was observed in the observational cohort studies [22–26], with a estimated risk difference of 0.07, 95%CI 0.04–0.10. The RCT and observational literature regarding glaucoma was particularly limited as was that addressing the effect of dose and duration of GC therapy. There were no studies addressing the impact of comorbidities known to be associated with increased risk of cataract or glaucoma, or the visual outcome of GC-induced cataract or glaucoma. PSCs often require surgery at an earlier stage than other cataract types [33] and whilst this has not been addressed in the context of GC use, it certainly raises concern and highlights the need for future studies in this area.

Although well described as side effects of GC use in review articles and books [34–38], cataracts have been reported in only three of the 28 RCTs comparing GC use and non-use in RA populations (in either arm of the study). Through comparison to general population cumulative incidence rates derived from the BMES, it is apparent there is significant under-reporting of cataracts in RA glucocorticoid clinical trials, making any estimates of GC-associated risk less reliable. Ideally, future RCTs comparing GC use to non-use would endeavour to accurately capture this information with regular ophthalmology assessments.

Whilst this review focussed specifically on studies in RA populations, it is interesting to consider how the extent of GC-associated risk compares with studies in other populations, and between the sub-types of cataract. In the BMES Australian general population there was an association between current GC use and PSCs (OR 4.11, 95%CI 1.67–10.08) and nuclear cataracts (OR 3.45, 95% CI 1.26–9.43) but not cortical cataracts (OR 1.08, 95%CI 0.44–2.64) [33]. In comparison, the Beaver Dam Eye Study found an increased incidence of cortical cataracts associated with GC use in the American general population (OR 2.59, 95%CI 1.45–4.62) but not PSCs (OR 1.27, 95%CI 0.42–3.86) or nuclear cataracts (OR 1.41, 95%CI 0.77–2.56) [39]. This is in contrast to other studies that have shown a strong association between GC use and PSCs in other populations including asthma and renal transplantation [33, 40–43]. Unfortunately, despite the observation that PSCs occurred with GC use reported in earlier cross-sectional studies, more recent RA studies have not reported specific cataract type.

### Impact of Dose and Duration of GC Therapy

The impact of dose and duration of GC therapy on the development of cataract and glaucoma remains unanswered. Whilst some of the observational studies in this review suggest an association between GC use and dose and duration of therapy, they do not adequately quantify the risk in a way that can be translated into clinical practice when discussing the risks and benefits of GC treatment for RA. Many of the observational studies that comment on dose and duration were insufficiently powered, or not appropriately designed, to determine an accurate association between different doses and durations of therapy. A striking association between GC use and the development of PSCs was described in early cross-sectional studies carried out in the 1960s, however an association with cataracts was not seen in more recent RCTs. Whilst under-reporting partly accounts for this, another possible explanation is that GC exposure (dose and duration of therapy) has declined over time with the increasing use of concurrent DMARDs, highlighting the need for this to be explored in future studies.

### Strengths and limitations

The main strength of this review is the systematic approach used to identify all relevant studies published since 1950 and the use of two reviewers to confirm eligibility criteria. The main limitation of this review is the small number of studies meeting eligibility criteria, making it impossible to draw meaningful conclusions. Although cross-sectional studies cannot assess causality, their inclusion allowed a comprehensive summary of the complete literature on this subject. The results are reported by study design and thus this does not detract from the study findings. The heterogeneity of studies was significant, particularly in regards to the methods used to detect cataracts and glaucoma. Only one of the RCTs and two of the cohort studies used examinations to detect cataracts, and the remainder relied on patient reports or case note review. There was additional heterogeneity in the definitions of GC exposure. This heterogeneity made it impossible to draw any quantitative conclusions regarding the risk of GC use and the development of cataract and glaucoma in RA patients. The observational studies had a high risk of bias, with only one [25] controlling for comorbidities known to be associated the development of cataract and glaucoma and potentially associated with GC exposure [44–46].

The current literature leaves many questions unanswered in regards to GC use and the development of cataract and glaucoma. Although RCTs often represent the highest level of evidence for efficacy, they are not ideal for examining long-term drug adverse effects. They also do not reflect the patterns of GC use seen in routine practice. In order to adequately address the questions in this review, a well designed observational study that is large enough and of sufficient duration to capture rare events is required. Ideally, such a study would be conducted in a real-life setting so that the impact of age and comorbidities could also be determined. Data on dose and duration of GC use would need to be accurately captured and appropriate methodologies employed to fully understand the interactions between dose, duration and recentness of treatment. Cataracts and glaucoma become increasingly prevalent with age and commonly occur without exposure to systemic GCs. They range in severity from asymptomatic to visually disabling, making it important to clearly define the clinical relevance of cataract/glaucoma in future studies.

### Conclusions

In conclusion, observational studies, but not RCTs, suggest an association between GC use and the development of cataracts in RA patients. This relationship has not been adequately quantified, nor have the effect of dose and duration of therapy, visual outcomes and the impact of comorbidities been addressed. Confidence in RCT and observational estimates are limited by under-reporting, and heterogeneity and bias, respectively. There is no robust literature regarding GC use and the development of glaucoma in RA patients. These findings highlight the need for future well conducted observational studies targeting the unanswered questions as well as improved capture of these outcomes in future RCTs.

## Supporting Information

S1 Table Search terms used for RCTs and Observational Studies in each of the databases. (DOCX)

S2 Table RCTs comparing GC use to non-use in RA and number of reported cataract and glaucoma. (DOCX)

S3 Table PRISMA 2009 Checklist. (DOC)
