# Supplementary material for: The Association between Systemic Glucocorticoid Use and the Risk of Cataract and Glaucoma in Patients with Rheumatoid Arthritis: A Systematic Review and Meta-Analysis
Source: PLoS One. 2016 Nov 15;11(11):e0166468. doi: 10.1371/journal.pone.0166468 (PMC5112962; doi:10.1371/journal.pone.0166468)
Supplement: S1 Table — (DOCX) [file pone.0166468.s001.docx]

**S1 Table. Search terms used for RCTs and Observational Studies in each of the databases.**

| **MEDLINE and EMBASE-**  **Observational studies and RCTs** | **Web of Science-**  **Observational studies and RCTs** |
| --- | --- |
| exp Adrenal Cortex Hormones/ | corticosteroid$ |
| corticosteroid$.mp. | glucocorticoid$ |
| glucocorticoid$.mp. | glucocorticosteroid$ |
| glucocorticosteroid$.mp. | steroid$ |
| steroid$.mp. | rheumatoid arthritis |
| exp Arthritis, Rheumatoid/ | inflammatory arthritis |
| Rheumatoid Arthritis.mp. | inflammatory polyarthritis |
| inflammatory arthritis.mp. |  |
| inflammatory polyarthritis.mp. |  |
| **MEDLINE and EMBASE-**  **Observational studies only** | **Web of Science-**  **Observational studies only** |
| exp Cataract/ | cataract$ |
| cataract.mp. | lens opac$ |
| lens opacity.mp. | glaucoma |
| (lens adj3 opacity).mp. | ocular hypertension |
| (lens adj3 opacification).mp. |  |
| glaucoma.mp. |  |
| exp Glaucoma/ |  |
| ocular adj3 hypertension).mp. |  |
