# Supplementary material for: The Association between Systemic Glucocorticoid Use and the Risk of Cataract and Glaucoma in Patients with Rheumatoid Arthritis: A Systematic Review and Meta-Analysis
Source: PLoS One. 2016 Nov 15;11(11):e0166468. doi: 10.1371/journal.pone.0166468 (PMC5112962; doi:10.1371/journal.pone.0166468)
Supplement: S2 Table — (DOCX) [file pone.0166468.s002.docx]

**S2 Table. RCTs comparing GC use to non-use in RA and number of reported cataract and glaucoma.**

| **First Author,**  **Year** | **Duration of study** | **Country** | **Mean Age***  **(%Female)** | **Arms of RCTs (n)** |  |  |  |
| --- | --- | --- | --- | --- | --- | --- | --- |
|  |  |  |  |  |  |  |  |
| Bakker(1),  2012 | 2 years | The Netherlands | 54  (60%) | MTX + PNL 10mg/d (117)  MTX + placebo (119) |  |  |  |
| Buttgereit(2),  2013 | 12 weeks | North America, Europe | 57.2  (84%) | MR-PNL 5mg/d (231)  Placebo (119) |  |  |  |
| Capell(3),  2004 | 2 years | United Kingdom | Median 56  (65%) | SSZ + PNL 7mg (84)  SSZ + placebo (83) |  |  |  |
| Chamberlain(4), 1976 | 2 years | United Kingdom | Range 28-75  (85%) | PNL 5mg (20)  PNL 3mg (10)  PNL 0mg (19) |  |  |  |
| Choy(5),  2005 | 2 years | United Kingdom | 58  (78%) | IM Depomedrone 120mg (48)  Saline placebo (43) |  |  |  |
| Choy(6),  2008 | 2 years | United Kingdom | 54  (70%) | MTX (117)  MTX + Ciclosporin (119)  MTX + PNL (115)  MTX + Ciclosporin + PNL (116) |  |  |  |
| Ciconelli(7),  1996 | 26 weeks | Brazil | 44  (100%) | SSZ + IV MP 5mg/kg  month 0,1 +2 (20)  SSZ + IV placebo (saline)  month 0, 1+2 (18) |  |  |  |
| Corkill(8),  1990 | 24 weeks | United Kingdom | 54  (64%) | Gold + IM MP 120mg  week 0,4 + 8 (35)  Gold + IM placebo (saline)  week 0,4 + 8 (24) |  |  |  |
| Durez(9),  2007 | 46 weeks | Belgium | 51  (66%) | MTX (14)  MTX + IV MP 1g (15)  MTX + infliximab (15) |  |  |  |
| Emery(10),  2006 | 24 weeks | International | 51  (81%) | RTX placebo + IV MP (42)  RTX placebo + IV MP + PO PNL (44)  RTX placebo + placebo (63)  RTX 500mg + IV MP (41)  RTX 500mg + IV MP + PO PNL (42)  RTX 500mg + placebo (41)  RTX 1000mg + IV MP (62)  RTX 1000mg + IV MP + PO PNL (65)  RTX 1000mg + placebo (55) |  |  |  |
| Gerlag(11),  2004 | 2 weeks | The Netherlands | 53  (62%) | PNL 60mg week 1,  40mg week 2 (10)  Placebo (11) |  |  |  |
| Gough(12),  1994 | 1 year | United Kingdom | 54  (80%) | SSZ + IM MP 120mg  week 0, 4 + 12 (11)  SSZ + IM placebo (saline)  week 0,4 + 12 (9) |  |  |  |
| Hansen(13),  1990 | 1 year | Denmark | 60  (73%) | DMARD (AZA or PEN) + IV MP  week 0,4,8,12,16 + 20 (31)  DMARD (AZA or PEN) + placebo (26) |  |  |  |
| Hansen(14),  1999 | 1 year | Denmark | 62  (NR) | DMARD + PNL (42)  DMARD only (34) |  |  |  |
| Kirwan(15),  1995 | 2 years | United Kingdom | 49  (64%) | PNL 7.5mg (61)  Placebo (67) |  |  |  |
| Kirwan(16),  2004 | 12 weeks | Belgium, Sweden, United Kingdom | 55  (71%) | Budesonide 3mg (37)  Budesonide 9mg (36)  PNL 7.5mg (39)  Placebo (31) |  |  |  |
| Laan(17),  1993 | 44 weeks | The Netherlands | 55  (70%) | Gold + PNL mean dose 7.5mg for 20 weeks (20)  Gold + placebo for 20 weeks (20) |  |  |  |
| Lee(18),  1973 | 2 weeks | United Kingdom | NR | PNL 5mg tds (45)  Aspirin 975mg qid (42)  Placebo qid (41) |  |  |  |
| Montecucco(19),  2012 | 1 year | Italy | 60  (64%) | MTX + PNL 12.5mg/d for 2 weeks then 6.25mg/d (96)  MTX alone (90) |  |  |  |
| Sheldon(20),  2003 | 4 weeks | United Kingdom | 57  (62%) | Budesonide CR (14)  Placebo (12) |  |  |  |
| Svensson(21),  2005 | 2 years | Sweden | 55  (64%) | DMARD + PNL 7.5mg (119)  DMARD alone, no placebo (131) |  |  |  |
| Todoerti(22),  2010 | 2 years | Italy | 60  (74%) | MTX + PNL 12.5mg for 2 weeks, then 6.25mg (105)  MTX + placebo (105) |  |  |  |
| van der Veen(23),  1993 | 1 year | The Netherlands | 56  (80%) | MTX + placebo (10)  MTX + PNL 100mg day 1,3+5 (10)  MTX + 1g IV MP day 1,3+5 (10) |  |  |  |
| van Everdingen(24),  2002 | 2 years | The Netherlands | 62  (64%) | PNL 10mg (40)  Placebo (41) |  |  |  |
| Verschueren(25),  2015 | 16 weeks | Belgium | 51  (79%) | MTX (47)  MTX + PNL 30mg tapered to 5mg at week 6 (43) |  |  |  |
| Wassenberg(26),  2005 | 2 years | Germany, Austria, Switzerland | 52  (70%) | Gold or MTX + PNL 5mg (93)  Gold or MTX + placebo (96) |  |  |  |
| Williams(27),  1982 | 6 weeks | United Kingdom | 56  (90%) | 1g IV MP (10)  Placebo (10) |  |  |  |
| Wong(28),  1990 | 24 weeks | Australia | 64  (38%) | Gold + 1g IV MP week 0,4+8 (20)  Gold + placebo (20) |  |  |  |

* mean age, unless otherwise stated

******Williams et al reported one open angle glaucoma but did not state whether it occurred in the GC or control group

GC=glucocorticoid, pyr= person years at risk, NR= not reported, No.=number, PNL=prednisolone, MR-PNL= modified release prednisolone, MTX= methotrexate, SSZ=sulfasalazine, MP=methylprednisolone, RTX=rituximab

1. Bakker MF, Jacobs JWG, Welsing PMJ, Verstappen SMM, Tekstra J, Ton E, et al. Low-dose prednisone inclusion in a methotrexate-based, tight control strategy for early rheumatoid arthritis: a randomized trial.[Summary for patients in Ann Intern Med. 2012 Mar 6;156(5):I18; PMID: 22393145]. Annals of internal medicine. 2012;156(5):329-39. PubMed PMID: 22393128.

2. Buttgereit F, Mehta D, Kirwan J, Szechinski J, Boers M, Alten RE, et al. Low-dose prednisone chronotherapy for rheumatoid arthritis: a randomised clinical trial (CAPRA-2). Annals of the rheumatic diseases. 2013;72(2):204-10. PubMed PMID: 22562974. Pubmed Central PMCID: PMC3553491.

3. Capell HA, Madhok R, Hunter JA, Porter D, Morrison E, Larkin J, et al. Lack of radiological and clinical benefit over two years of low dose prednisolone for rheumatoid arthritis: results of a randomised controlled trial. Annals of the rheumatic diseases. 2004;63(7):797-803. PubMed PMID: 15194574. Pubmed Central PMCID: PMC1755058.

4. Chamberlain MA, Keenan J. The effect of low doses of prednisolone compared with placebo on function and on the hypothalamic pituitary adrenal axis in patients with rheumatoid arthritis. Rheumatology and rehabilitation. 1976 Feb;15(1):17-23. PubMed PMID: 766142. Epub 1976/02/01. eng.

5. Choy EH, Kingsley GH, Khoshaba B, Pipitone N, Scott DL, Intramuscular Methylprednisolone Study G. A two year randomised controlled trial of intramuscular depot steroids in patients with established rheumatoid arthritis who have shown an incomplete response to disease modifying antirheumatic drugs. Annals of the rheumatic diseases. 2005 Sep;64(9):1288-93. PubMed PMID: 15760929. Pubmed Central PMCID: 1755652. Epub 2005/03/12. eng.

6. Choy EH, Smith CM, Farewell V, Walker D, Hassell A, Chau L, et al. Factorial randomised controlled trial of glucocorticoids and combination disease modifying drugs in early rheumatoid arthritis. Annals of the rheumatic diseases. 2008 May;67(5):656-63. PubMed PMID: 17768173. Epub 2007/09/05. eng.

7. Ciconelli RM, Ferraz MB, Visioni RA, Oliveira LM, Atra E. A randomized double-blind controlled trial of sulphasalazine combined with pulses of methylprednisolone or placebo in the treatment of rheumatoid arthritis. British journal of rheumatology. 1996;35(2):150-4. PubMed PMID: 8612028.

8. Corkill MM, Kirkham BW, Chikanza IC, Gibson T, Panayi GS. Intramuscular depot methylprednisolone induction of chrysotherapy in rheumatoid arthritis: a 24-week randomized controlled trial. British journal of rheumatology. 1990;29(4):274-9. PubMed PMID: 2198977.

9. Durez P, Malghem J, Nzeusseu Toukap A, Depresseux G, Lauwerys BR, Westhovens R, et al. Treatment of early rheumatoid arthritis: a randomized magnetic resonance imaging study comparing the effects of methotrexate alone, methotrexate in combination with infliximab, and methotrexate in combination with intravenous pulse methylprednisolone. Arthritis and rheumatism. 2007 Dec;56(12):3919-27. PubMed PMID: 18050189. Epub 2007/12/01. eng.

10. Emery P, Fleischmann R, Filipowicz-Sosnowska A, Schechtman J, Szczepanski L, Kavanaugh A, et al. The efficacy and safety of rituximab in patients with active rheumatoid arthritis despite methotrexate treatment: results of a phase IIB randomized, double-blind, placebo-controlled, dose-ranging trial. Arthritis & Rheumatism. 2006;54(5):1390-400. PubMed PMID: 16649186.

11. Gerlag DM, Haringman JJ, Smeets TJ, Zwinderman AH, Kraan MC, Laud PJ, et al. Effects of oral prednisolone on biomarkers in synovial tissue and clinical improvement in rheumatoid arthritis. Arthritis and rheumatism. 2004 Dec;50(12):3783-91. PubMed PMID: 15593225. Epub 2004/12/14. eng.

12. Gough A, Sheeran T, Arthur V, Panayi G, Emery P. Adverse interaction between intramuscular methylprednisolone and sulphasalazine in patients with early rheumatoid arthritis. A pilot study. Scandinavian Journal of Rheumatology. 1994;23(1):46-8. PubMed PMID: 7906428.

13. Hansen TM, Kryger P, Elling H, Haar D, Kreutzfeldt M, Ingeman-Nielsen MW, et al. Double blind placebo controlled trial of pulse treatment with methylprednisolone combined with disease modifying drugs in rheumatoid arthritis. Bmj. 1990;301(6746):268-70. PubMed PMID: 2202458. Pubmed Central PMCID: PMC1663457.

14. Hansen M, Podenphant J, Florescu A, Stoltenberg M, Borch A, Kluger E, et al. A randomised trial of differentiated prednisolone treatment in active rheumatoid arthritis. Clinical benefits and skeletal side effects. Annals of the rheumatic diseases. 1999;58(11):713-8. PubMed PMID: 10531077. Pubmed Central PMCID: PMC1752793.

15. Kirwan JR. The effect of glucocorticoids on joint destruction in rheumatoid arthritis. The Arthritis and Rheumatism Council Low-Dose Glucocorticoid Study Group. The New England journal of medicine. 1995 Jul 20;333(3):142-6. PubMed PMID: 7791815. Epub 1995/07/20. eng.

16. Kirwan JR, Hallgren R, Mielants H, Wollheim F, Bjorck E, Persson T, et al. A randomised placebo controlled 12 week trial of budesonide and prednisolone in rheumatoid arthritis. Annals of the rheumatic diseases. 2004 Jun;63(6):688-95. PubMed PMID: 15140776. Pubmed Central PMCID: 1755023. Epub 2004/05/14. eng.

17. Laan R, Vanriel P, Vandeputte LBA, Vanerning L, Vanthof MA, Lemmens JAM. LOW-DOSE PREDNISONE INDUCES RAPID REVERSIBLE AXIAL BONE LOSS IN PATIENTS WITH RHEUMATOID-ARTHRITIS - A RANDOMIZED, CONTROLLED-STUDY. Annals of internal medicine. 1993 Nov;119(10):963-8. PubMed PMID: WOS:A1993MG67200001.

18. Lee P, Jasani MK, Dick WC, Buchanan WW. Evaluation of a functional index in rheumatoid arthritis. Scand J Rheumatol. 1973;2(2):71-7. PubMed PMID: 4584370. Epub 1973/01/01. eng.

19. Montecucco C, Todoerti M, Sakellariou G, Scire CA, Caporali R. Low-dose oral prednisone improves clinical and ultrasonographic remission rates in early rheumatoid arthritis: results of a 12-month open-label randomised study. Arthritis research & therapy. 2012;14(3):R112. PubMed PMID: 22584017. Pubmed Central PMCID: PMC3446489.

20. Sheldon P. Ileum-targeted steroid therapy in rheumatoid arthritis: double-blind, placebo-controlled trial of controlled-release budesonide. Rheumatology international. 2003 Jul;23(4):154-8. PubMed PMID: 12856138. Epub 2003/07/12. eng.

21. Svensson B, Boonen A, Albertsson K, van der Heijde D, Keller C, Hafstrom I. Low-dose prednisolone in addition to the initial disease-modifying antirheumatic drug in patients with early active rheumatoid arthritis reduces joint destruction and increases the remission rate: a two-year randomized trial. Arthritis and rheumatism. 2005 Nov;52(11):3360-70. PubMed PMID: 16255010. Epub 2005/10/29. eng.

22. Todoerti M, Scire CA, Boffini N, Bugatti S, Montecucco C, Caporali R. Early disease control by low-dose prednisone comedication may affect the quality of remission in patients with early rheumatoid arthritis. Annals of the New York Academy of Sciences. 2010;1193:139-45. PubMed PMID: 20398020.

23. van der Veen MJ, Bijlsma JW. The effect of methylprednisolone pulse therapy on methotrexate treatment of rheumatoid arthritis. Clinical rheumatology. 1993;12(4):500-5. PubMed PMID: 8124913.

24. van Everdingen AA, Jacobs JWG, Siewertsz Van Reesema DR, Bijlsma JWJ. Low-dose prednisone therapy for patients with early active rheumatoid arthritis: clinical efficacy, disease-modifying properties, and side effects: a randomized, double-blind, placebo-controlled clinical trial.[Summary for patients in Ann Intern Med. 2002 Jan 1;136(1):I-26; PMID: 11777372]. Annals of internal medicine. 2002;136(1):1-12. PubMed PMID: 11777359.

25. Verschueren P, De Cock D, Corluy L, Joos R, Langenaken C, Taelman V, et al. Methotrexate in combination with other DMARDs is not superior to methotrexate alone for remission induction with moderate-to-high-dose glucocorticoid bridging in early rheumatoid arthritis after 16 weeks of treatment: the CareRA trial. Annals of the rheumatic diseases. 2015 Jan;74(1):27-34. PubMed PMID: 25359382.

26. Wassenberg S, Rau R, Steinfeld P, Zeidler H. Very low-dose prednisolone in early rheumatoid arthritis retards radiographic progression over two years: a multicenter, double-blind, placebo-controlled trial. Arthritis and rheumatism. 2005 Nov;52(11):3371-80. PubMed PMID: 16255011. Epub 2005/10/29. eng.

27. Williams IA, Baylis EM, Shipley ME. A double-blind placebo-controlled trial of methylprednisolone pulse therapy in active rheumatoid disease. Lancet. 1982 Jul 31;2(8292):237-40. PubMed PMID: 6124671. Epub 1982/07/31. eng.

28. Wong CS, Champion G, Smith MD, Soden M, Wetherall M, Geddes RA, et al. Does steroid pulsing influence the efficacy and toxicity of chrysotherapy? A double blind, placebo controlled study. Annals of the rheumatic diseases. 1990 Jun;49(6):370-2. PubMed PMID: 2116773. Pubmed Central PMCID: 1004102. Epub 1990/06/01. eng.
